# Supplementary material for: Modeling glioblastoma heterogeneity as a dynamic network of cell states
Source: Mol Syst Biol. 2021 Sep 16;17(9):e10105. doi: 10.15252/msb.202010105 (PMC8444284; doi:10.15252/msb.202010105)
Supplement: Supplementary file 6 — Source Data for Figure 5 [file MSB-17-e10105-s004.zip › Figure5A_sourcedata/GSEA_3017/nosigdb_stateE.GseaPreranked.1622795031093/RICHARDS_NOWAKOWSKI_SCIENCE_2017_ORG_UPREG.html]

Details for gene set RICHARDS\_NOWAKOWSKI\_SCIENCE\_2017\_ORG\_UPREG[GSEA]

|  || Dataset | state63017 |
| Phenotype | NoPhenotypeAvailable |
| Upregulated in class | na\_pos |
| GeneSet | RICHARDS\_NOWAKOWSKI\_SCIENCE\_2017\_ORG\_UPREG |
| Enrichment Score (ES) | 0.48560303 |
| Normalized Enrichment Score (NES) | 4.116241 |
| Nominal p-value | 0.0 |
| FDR q-value | 0.0 |
| FWER p-Value | 0.0 |
Table: GSEA Results Summary

  

Fig 1: Enrichment plot: RICHARDS\_NOWAKOWSKI\_SCIENCE\_2017\_ORG\_UPREG      
 Profile of the Running ES Score & Positions of GeneSet Members on the Rank Ordered List

  

| PROBE | GENE SYMBOL | GENE\_TITLE | RANK IN GENE LIST | RANK METRIC SCORE | RUNNING ES | CORE ENRICHMENT || 1 | PMP22 |  |  | 0 | 1.393 | 0.0208 | Yes |
| 2 | GFAP |  |  | 1 | 1.383 | 0.0415 | Yes |
| 3 | CYR61 |  |  | 3 | 1.169 | 0.0578 | Yes |
| 4 | IGFBP5 |  |  | 4 | 1.015 | 0.0729 | Yes |
| 5 | SPARCL1 |  |  | 7 | 0.963 | 0.0848 | Yes |
| 6 | LRRN3 |  |  | 10 | 0.943 | 0.0964 | Yes |
| 7 | FABP7 |  |  | 11 | 0.920 | 0.1102 | Yes |
| 8 | CD44 |  |  | 14 | 0.806 | 0.1197 | Yes |
| 9 | ANXA2 |  |  | 18 | 0.733 | 0.1268 | Yes |
| 10 | TTYH1 |  |  | 19 | 0.731 | 0.1378 | Yes |
| 11 | CNN3 |  |  | 24 | 0.702 | 0.1432 | Yes |
| 12 | LGALS3 |  |  | 25 | 0.697 | 0.1536 | Yes |
| 13 | IFITM3 |  |  | 26 | 0.682 | 0.1638 | Yes |
| 14 | MSMO1 |  |  | 28 | 0.648 | 0.1723 | Yes |
| 15 | CYP51A1 |  |  | 30 | 0.637 | 0.1805 | Yes |
| 16 | TAGLN2 |  |  | 32 | 0.621 | 0.1886 | Yes |
| 17 | RAB31 |  |  | 35 | 0.598 | 0.1950 | Yes |
| 18 | HMGCS1 |  |  | 37 | 0.593 | 0.2026 | Yes |
| 19 | NEDD9 |  |  | 38 | 0.590 | 0.2114 | Yes |
| 20 | CALD1 |  |  | 41 | 0.580 | 0.2175 | Yes |
| 21 | C1orf61 |  |  | 43 | 0.553 | 0.2245 | Yes |
| 22 | ARID5B |  |  | 47 | 0.533 | 0.2287 | Yes |
| 23 | GPC1 |  |  | 50 | 0.530 | 0.2341 | Yes |
| 24 | UBL3 |  |  | 53 | 0.514 | 0.2392 | Yes |
| 25 | PEG10 |  |  | 54 | 0.506 | 0.2468 | Yes |
| 26 | F3 |  |  | 55 | 0.506 | 0.2544 | Yes |
| 27 | MRC2 |  |  | 56 | 0.505 | 0.2620 | Yes |
| 28 | SLC1A3 |  |  | 66 | 0.472 | 0.2576 | Yes |
| 29 | SRGAP3 |  |  | 67 | 0.467 | 0.2646 | Yes |
| 30 | NACC2 |  |  | 68 | 0.464 | 0.2715 | Yes |
| 31 | HSPA5 |  |  | 73 | 0.449 | 0.2732 | Yes |
| 32 | SERPINB6 |  |  | 77 | 0.440 | 0.2760 | Yes |
| 33 | PDPN |  |  | 79 | 0.438 | 0.2812 | Yes |
| 34 | PPP1CB |  |  | 80 | 0.438 | 0.2878 | Yes |
| 35 | DOK5 |  |  | 81 | 0.437 | 0.2943 | Yes |
| 36 | SIPA1L1 |  |  | 84 | 0.429 | 0.2982 | Yes |
| 37 | CST3 |  |  | 86 | 0.425 | 0.3033 | Yes |
| 38 | NDFIP1 |  |  | 91 | 0.416 | 0.3044 | Yes |
| 39 | NES |  |  | 92 | 0.414 | 0.3106 | Yes |
| 40 | EFNB2 |  |  | 94 | 0.411 | 0.3155 | Yes |
| 41 | SHROOM3 |  |  | 95 | 0.410 | 0.3217 | Yes |
| 42 | EZR |  |  | 96 | 0.410 | 0.3278 | Yes |
| 43 | STK17A |  |  | 97 | 0.408 | 0.3339 | Yes |
| 44 | ATF3 |  |  | 101 | 0.397 | 0.3360 | Yes |
| 45 | GPM6B |  |  | 105 | 0.391 | 0.3381 | Yes |
| 46 | CD99 |  |  | 107 | 0.389 | 0.3426 | Yes |
| 47 | CHN1 |  |  | 112 | 0.373 | 0.3431 | Yes |
| 48 | LGALS1 |  |  | 113 | 0.368 | 0.3486 | Yes |
| 49 | CLU |  |  | 119 | 0.364 | 0.3477 | Yes |
| 50 | FNDC3B |  |  | 123 | 0.354 | 0.3492 | Yes |
| 51 | AHNAK |  |  | 125 | 0.351 | 0.3532 | Yes |
| 52 | FHL1 |  |  | 126 | 0.351 | 0.3585 | Yes |
| 53 | SEMA6A |  |  | 130 | 0.348 | 0.3599 | Yes |
| 54 | ARL6IP5 |  |  | 134 | 0.345 | 0.3612 | Yes |
| 55 | CETN2 |  |  | 145 | 0.338 | 0.3536 | Yes |
| 56 | NUDT4 |  |  | 146 | 0.337 | 0.3586 | Yes |
| 57 | NEK6 |  |  | 147 | 0.337 | 0.3637 | Yes |
| 58 | GATM |  |  | 149 | 0.335 | 0.3674 | Yes |
| 59 | TUBB2A |  |  | 157 | 0.327 | 0.3634 | Yes |
| 60 | MVD |  |  | 158 | 0.327 | 0.3683 | Yes |
| 61 | ZFP36L2 |  |  | 162 | 0.324 | 0.3693 | Yes |
| 62 | FZD7 |  |  | 164 | 0.323 | 0.3729 | Yes |
| 63 | SQLE |  |  | 168 | 0.319 | 0.3739 | Yes |
| 64 | DDAH1 |  |  | 170 | 0.317 | 0.3774 | Yes |
| 65 | JUN |  |  | 172 | 0.316 | 0.3808 | Yes |
| 66 | MBNL2 |  |  | 173 | 0.316 | 0.3855 | Yes |
| 67 | NLGN4X |  |  | 177 | 0.312 | 0.3864 | Yes |
| 68 | FADS2 |  |  | 181 | 0.310 | 0.3872 | Yes |
| 69 | LRRC17 |  |  | 182 | 0.310 | 0.3919 | Yes |
| 70 | SLC35F1 |  |  | 183 | 0.309 | 0.3965 | Yes |
| 71 | PLIN3 |  |  | 192 | 0.301 | 0.3908 | Yes |
| 72 | SLC25A18 |  |  | 195 | 0.300 | 0.3928 | Yes |
| 73 | GLUL |  |  | 196 | 0.300 | 0.3973 | Yes |
| 74 | BICD1 |  |  | 198 | 0.297 | 0.4005 | Yes |
| 75 | CAST |  |  | 202 | 0.294 | 0.4010 | Yes |
| 76 | DKK3 |  |  | 205 | 0.293 | 0.4029 | Yes |
| 77 | SAT1 |  |  | 206 | 0.291 | 0.4072 | Yes |
| 78 | GNG11 |  |  | 210 | 0.289 | 0.4078 | Yes |
| 79 | GNG12 |  |  | 212 | 0.287 | 0.4108 | Yes |
| 80 | MEST |  |  | 215 | 0.285 | 0.4125 | Yes |
| 81 | CHPT1 |  |  | 216 | 0.284 | 0.4168 | Yes |
| 82 | PLEKHG1 |  |  | 217 | 0.284 | 0.4210 | Yes |
| 83 | IDH1 |  |  | 218 | 0.284 | 0.4253 | Yes |
| 84 | FERMT2 |  |  | 219 | 0.283 | 0.4295 | Yes |
| 85 | TMEM47 |  |  | 222 | 0.282 | 0.4312 | Yes |
| 86 | SAMD4A |  |  | 224 | 0.281 | 0.4341 | Yes |
| 87 | SDC2 |  |  | 226 | 0.280 | 0.4371 | Yes |
| 88 | NPAS3 |  |  | 227 | 0.280 | 0.4412 | Yes |
| 89 | DAG1 |  |  | 228 | 0.279 | 0.4454 | Yes |
| 90 | GUK1 |  |  | 231 | 0.277 | 0.4470 | Yes |
| 91 | TMED9 |  |  | 234 | 0.272 | 0.4485 | Yes |
| 92 | RNF180 |  |  | 235 | 0.272 | 0.4526 | Yes |
| 93 | HS6ST1 |  |  | 237 | 0.269 | 0.4554 | Yes |
| 94 | ITGB8 |  |  | 239 | 0.268 | 0.4581 | Yes |
| 95 | QKI |  |  | 241 | 0.267 | 0.4608 | Yes |
| 96 | CHMP2A |  |  | 243 | 0.266 | 0.4636 | Yes |
| 97 | GPC4 |  |  | 246 | 0.264 | 0.4650 | Yes |
| 98 | HMGCR |  |  | 251 | 0.261 | 0.4638 | Yes |
| 99 | INSIG1 |  |  | 252 | 0.261 | 0.4677 | Yes |
| 100 | GSTM3 |  |  | 257 | 0.259 | 0.4665 | Yes |
| 101 | SLC4A4 |  |  | 258 | 0.259 | 0.4704 | Yes |
| 102 | LEPROT |  |  | 259 | 0.259 | 0.4742 | Yes |
| 103 | ATP1B2 |  |  | 260 | 0.258 | 0.4781 | Yes |
| 104 | WWTR1 |  |  | 263 | 0.256 | 0.4794 | Yes |
| 105 | FDFT1 |  |  | 266 | 0.252 | 0.4806 | Yes |
| 106 | MOXD1 |  |  | 268 | 0.252 | 0.4831 | Yes |
| 107 | EVA1C |  |  | 270 | 0.250 | 0.4856 | Yes |
| 108 | MICU1 |  |  | 279 | -0.253 | 0.4792 | No |
| 109 | PPP2R2D |  |  | 284 | -0.253 | 0.4779 | No |
| 110 | ZFP36L1 |  |  | 287 | -0.255 | 0.4792 | No |
| 111 | ACBD5 |  |  | 307 | -0.259 | 0.4590 | No |
| 112 | FAT3 |  |  | 311 | -0.261 | 0.4591 | No |
| 113 | CA12 |  |  | 325 | -0.263 | 0.4465 | No |
| 114 | CNTLN |  |  | 326 | -0.264 | 0.4505 | No |
| 115 | HSPD1 |  |  | 329 | -0.264 | 0.4519 | No |
| 116 | KNTC1 |  |  | 352 | -0.269 | 0.4280 | No |
| 117 | TRIOBP |  |  | 388 | -0.278 | 0.3878 | No |
| 118 | SLC12A2 |  |  | 389 | -0.279 | 0.3919 | No |
| 119 | RECQL |  |  | 390 | -0.279 | 0.3961 | No |
| 120 | HSPA8 |  |  | 392 | -0.280 | 0.3990 | No |
| 121 | CTBP2 |  |  | 393 | -0.280 | 0.4032 | No |
| 122 | DERA |  |  | 402 | -0.284 | 0.3973 | No |
| 123 | MAGI1 |  |  | 413 | -0.287 | 0.3889 | No |
| 124 | PPA1 |  |  | 420 | -0.289 | 0.3856 | No |
| 125 | LPAR4 |  |  | 433 | -0.294 | 0.3748 | No |
| 126 | NEDD1 |  |  | 491 | -0.311 | 0.3071 | No |
| 127 | GGH |  |  | 504 | -0.314 | 0.2966 | No |
| 128 | BARD1 |  |  | 510 | -0.318 | 0.2950 | No |
| 129 | SESN3 |  |  | 526 | -0.322 | 0.2808 | No |
| 130 | RHEB |  |  | 528 | -0.323 | 0.2843 | No |
| 131 | CSPG5 |  |  | 535 | -0.328 | 0.2816 | No |
| 132 | JADE1 |  |  | 543 | -0.329 | 0.2776 | No |
| 133 | ETV1 |  |  | 563 | -0.341 | 0.2586 | No |
| 134 | CEP128 |  |  | 565 | -0.342 | 0.2625 | No |
| 135 | GGCT |  |  | 598 | -0.356 | 0.2272 | No |
| 136 | COMMD4 |  |  | 601 | -0.358 | 0.2300 | No |
| 137 | PRTFDC1 |  |  | 613 | -0.364 | 0.2215 | No |
| 138 | HNRNPF |  |  | 621 | -0.369 | 0.2181 | No |
| 139 | DNER |  |  | 640 | -0.382 | 0.2010 | No |
| 140 | MRPS6 |  |  | 641 | -0.382 | 0.2067 | No |
| 141 | PHIP |  |  | 643 | -0.384 | 0.2112 | No |
| 142 | ZEB1 |  |  | 653 | -0.396 | 0.2057 | No |
| 143 | EGR1 |  |  | 656 | -0.398 | 0.2091 | No |
| 144 | GINS2 |  |  | 658 | -0.399 | 0.2138 | No |
| 145 | DCK |  |  | 659 | -0.400 | 0.2198 | No |
| 146 | RHNO1 |  |  | 664 | -0.406 | 0.2208 | No |
| 147 | ARC |  |  | 667 | -0.408 | 0.2244 | No |
| 148 | PGAM1 |  |  | 670 | -0.410 | 0.2280 | No |
| 149 | RPA2 |  |  | 688 | -0.424 | 0.2127 | No |
| 150 | E2F3 |  |  | 700 | -0.431 | 0.2052 | No |
| 151 | PSAT1 |  |  | 720 | -0.448 | 0.1878 | No |
| 152 | MIS18A |  |  | 750 | -0.483 | 0.1582 | No |
| 153 | KIF5B |  |  | 770 | -0.515 | 0.1418 | No |
| 154 | DBI |  |  | 776 | -0.526 | 0.1433 | No |
| 155 | DSEL |  |  | 794 | -0.563 | 0.1302 | No |
| 156 | CREM |  |  | 820 | -0.611 | 0.1076 | No |
| 157 | FAM111A |  |  | 858 | -0.727 | 0.0715 | No |
| 158 | HIST1H1C |  |  | 936 | -1.220 | -0.0079 | No |
| 159 | FABP5 |  |  | 939 | -1.294 | 0.0089 | No |
Table: GSEA details [plain text format]

  

Fig 2: RICHARDS\_NOWAKOWSKI\_SCIENCE\_2017\_ORG\_UPREG: Random ES distribution      
 Gene set null distribution of ES for **RICHARDS\_NOWAKOWSKI\_SCIENCE\_2017\_ORG\_UPREG**
